# Annual Announcement of the Jefferson Medical College of Philadelphia

**Published:** 1846-09

**Authors:** 


*Annual Announcement of the Jefferson Medical College of Philadelphia, Session of* 1846–'47.

It would seem from the above announcement, the receipt of which we take pleasure in acknowledging, that the Jefferson Medical College was never in a more flourishing condition than at present. So rapidly have the number of students attending the lectures of this institution increased, that the trustees and faculty have found it necessary to enlarge their lecture rooms. There were four hundred and nine in attendance upon the lectures of the last session, of whom one hundred and sixteen graduated.—*Balt. Ed.*

